# Elucidation of *Bifidobacterium* isolates from human milk and feces: investigating their anti-inflammatory effects on raw 264.7 via NF-κB signaling pathway

**DOI:** 10.3389/fmicb.2026.1763675

**Published:** 2026-03-16

**Authors:** Kexin Zhang, Mei-Suet Law, Tim-Fat Shum, Jiachi Chiou

**Affiliations:** 1 Department of Food Science and Nutrition, The Hong Kong Polytechnic University, Kowloon, Hong Kong SAR, China; 2 Research Institute for Future Food, The Hong Kong Polytechnic University, Kowloon, Hong Kong SAR, China

**Keywords:** *Bifidobacterium*, breastmilk, macrophage proliferation, NF-κB signaling pathway, *S. aureus* EVs

## Abstract

This study aimed to identify potent strains from seven *Bifidobacterium* isolates by evaluating their ability to counteract inflammation triggered by *Staphylococcus aureus*-derived extracellular vesicles (SA-EVs). Characteristics such as tolerance to simulated gastric and intestinal juice, adhesion/competitive adhesion, antimicrobial activity, and anti-inflammatory effects were investigated. Generation time of tested isolates ranged from 112.27 to 135.24 min, except for *Bifidobacterium adolescentis* AC19 (64.56 min) and *Bifidobacterium longum* AC18 (208.20 min). Seven strains, except *B. longum* CICC6186, remained viable at pH 3.0 (maximal reduction from 10^8^ to 10^7^ CFU/mL), yet were unable to withstand 3.0 g/L bile salts for 3 h (reduced from 10^8^ to 0 CFU/mL). They demonstrated good competitive adhesion ability against *Staphylococcus aureus* and *Escherichia coli* on Caco-2 cells with an average of 55.86% and 57.41% reduction of bacteria/cell, respectively, and showed strong antimicrobial ability against both Gram-positive and Gram-negative bacteria, possibly via production of acid and bacteriocins. Heat-inactivated *Bifidobacterium* (10^6^ CFU/mL) inhibited the inflammation triggered by SA-EVs, most likely by suppressing RAW-BLUE™ proliferation into M1 and TNF-α secretion, in turn affecting the activation of the NF-κB signaling pathway. *B. longum* AC15 showed overall outstanding capabilities amongst all, especially the adhesion ability to enterocytes, tolerance to acid and bile salt, and anti-inflammatory ability on macrophages.

## Graphical abstract

**Figure:**
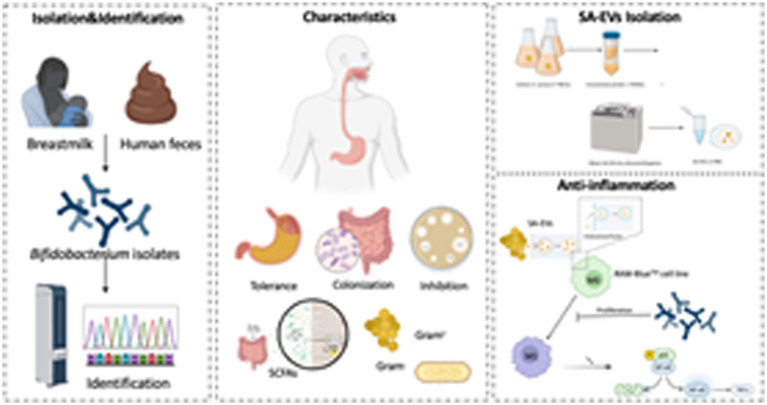

## Introduction

Bifidobacteria are Gram-positive, non-spore-forming, strictly anaerobic, pleomorphic heterofermentative bacteria that normally live in the gastrointestinal (GI) tract of humans and other animals (Saturio et al., 2021). These bacteria are constant companions throughout the human lifespan, yet their prevalence and dominant species undergo dynamic shifts with age. In early life, species such as *Bifidobacterium breve*, *Bifidobacterium bifidum,* and *Bifidobacterium longum* subsp*. infantis* are prevalent in the infants’ GI tract due to their genetic machinery of utilizing human milk oligosaccharides (HMOs) and the supporting relationship of cross-feeding (Xiao et al., 2024). With the gradual introduction of solid food, *Bifidobacterium longum* subsp. *longum*, *Bifidobacterium adolescentis*, and *Bifidobacterium catenulatum* become predominant in the gastrointestinal tract, as they are adapted to digesting dietary fiber (Arboleya et al., 2016; Turroni et al., 2019; Tarracchini et al., 2021). Succession of different *Bifidobacterium* (sub)species is not merely a change in taxonomy but reflects a functional specialization, where different species exhibit distinct capabilities that benefit host health through diverse mechanisms.

Bifidobacteria have attracted the interest of scientists since their initial discovery due to their wide-ranging physiological benefits for the host. One key mechanism of protection is their ability to adhere to intestinal receptors, proliferate, and saturate available physical niches, thereby sterically hindering the subsequent attachment of enteropathogens like *Escherichia coli*, *Salmonella enterica* serovar Typhimurium, or *Listeria monocytogenes* (Westermann et al., 2016). The Caco-2 cell line is the primary *in vitro* model for studying bacterial adhesion due to its unique differentiation into a polarized monolayer, which forms tight junctions and a functional microvilli brush border, closely mimicking the structure and function of mature small intestinal enterocytes (Fekete et al., 2024). Beyond competitive exclusion, bifidobacteria actively fortify intestinal barrier integrity. Certain strains upregulate the expression of tight junction proteins, such as ZO-1, occludin, and claudins, which strengthens the transepithelial electrical resistance (TEER) (Abdulqadir et al., 2023). This enhancement reduces paracellular permeability and helps prevent the translocation of pathogens across the monolayer.

Short-chain fatty acids (SCFAs) are not just byproducts of the fermentation of bifidobacteria but are essential bioactive mediators that orchestrate host–microbiota symbiosis (Akhtar et al., 2022). These key bioactive metabolites, such as acetate, propionate, butyrate, valerate, iso-valerate, and iso-butyrate, serve as important signaling molecules and energy sources, exerting broad systemic effects on the host. As the primary SCFA produced by bifidobacteria, acetate enhances the defensive capabilities of the intestinal epithelial cells, shielding the host from invading pathogens (Fukuda et al., 2012). Also, it lowers the luminal pH, creating a hostile environment for pH-sensitive pathogens, such as *Salmonella* and *E. coli* (Li et al., 2022). Butyrate is a typical energy source that supplies 60%–70% of the energy requirement of colonocytes, although bifidobacteria are not major producers (Hodgkinson et al., 2023). Apart from those well-known functions, SCFAs act as signaling molecules by activating G protein-coupled receptors (GPCRs), such as FFAR2 and FFAR3, to regulate hormone secretion, immune responses, and blood pressure. Moreover, they directly inhibit histone deacetylases (HDACs), leading to epigenetic modulation of gene expression involved in inflammation, cell proliferation, and metabolism (Rekha et al., 2024).

Notably, bifidobacteria demonstrate anti-inflammatory potential, with a key mechanism being the direct modulation of macrophage polarization and function. Macrophages are central orchestrators of the immune response, capable of polarizing into a pro-inflammatory (M1) or an anti-inflammatory, reparative (M2) phenotype (Chen et al., 2023). Metabolites, such as lactic acid and SCFAs secreted by bifidobacteria, can shift the balance toward the M2 phenotype, thereby alleviating inflammation. *Staphylococcus aureus* (*S. aureus*) colonization has been associated with many health-related infections, such as lower respiratory tract, blood, and peritoneal or intra-abdominal infections (Piewngam and Otto, 2024). Moreover, *S. aureus* is frequently implicated in mild to moderate skin infections in the community (Bennett et al., 2025). It secretes a variety of enterotoxins and other toxins in an extracellular vesicle form (SA-EVs), which causes inflammatory responses and activates pro-inflammatory cells like macrophages (Chen et al., 2022). EVs are recognized as nanocarriers that could transport virulence factors to the host tissues and play a significant role in the interactions between bacteria and hosts (Tartaglia et al., 2018). SA-EVs are strongly associated with the development of many inflammatory diseases, particularly atopic dermatitis (Hong et al., 2011). Despite the well-documented anti-inflammatory properties of bifidobacteria and the established role of SA-EVs in driving inflammation, whether bifidobacteria can directly attenuate SA-EV-induced inflammatory responses has never been investigated to date.

Given the distinct functional attributes of different bifidobacterial species and strains, targeted characterization is essential to identify strains suited for specific applications. This study aims to (1) perform an *in vitro* comparative characterization of human-derived bifidobacteria and (2) evaluate and identify potential *Bifidobacterium* strains capable of surviving gastrointestinal transit and exhibiting anti-inflammatory functions. The findings will establish a preliminary basis for subsequent investigations into the capacity of *Bifidobacterium* strains to attenuate inflammation induced by SA-EVs.

## Methods

### Isolation, optimization of culture conditions, and identification of *Bifidobacterium* strains

A cohort of nine participants was recruited for specimen collection. Fresh fecal samples were obtained from six adult individuals (comprising three women and three men) aged 26–33 years, as well as from one child aged 5 years. Additionally, fresh breastmilk samples were provided by three lactating mothers from Hong Kong during the postnatal period. From these samples, six *Bifidobacterium* isolates were successfully isolated by using five different broths solidified with 1.5% agar, such as Tryptic Soy broth (TSB) (Cat#HB8570-1), BL broth (Cat#HB0395), *Bifidobacterium*-selective media (BSM) broth (Cat#HB0396-1) purchased from Hope Bio-Technology (Qingdao, China), Reinforced Clostridial Medium (RCM) (Cat#CM0149B, OXOID Ltd., Ireland, UK), and De Man, Rogosa & Sharpe (MRS) (Cat#LA4360, Solarbio, Beijing, China). These agars were supplemented with 100 mg/L of mupirocin (Cat#T1465-100MG, TargetMol, Boston, USA) and 90 mg/L of 8-hydroxyquinoline (Cat#H6878-100 g, Sigma Aldrich, St. Louis, Missouri, USA) as selective agents for *Bifidobacterium* isolation. Mupirocin is a selective inhibitor of Gram-positive bacteria, particularly most lactic acid bacteria and *Staphylococcus* (Sun et al., 2024), while 8-hydroxyquinoline provides broad-spectrum backup against organisms that mupirocin misses, including Gram-negative bacteria, yeasts, and molds (Saadeh et al., 2020).

*Bifidobacterium* isolates included in the study belonged to three species: *B. longum*, *B. adolescentis,* and *B. pseudocatenulatum*. A 200 μL aliquot of each *Bifidobacterium* stock was inoculated into 10 mL RCM broth. Cultures were then incubated anaerobically at 37 °C in an atmosphere of 5% CO_2_, 5% H_2_, and 90% N_2_ using an anaerobic chamber (Concept 400, Baker Ruskinn, Troisdorf, Germany). All the experiments were conducted with three independent biological replicates. Taxonomic identification in Table 1 was confirmed by two methodologies, such as 16S rRNA sequencing using the universal pair of primers 27F (5′-AGAGTTTGATCCTGGCTCAG-3′) and 1492R (5′-TACGGCTACCTTGTTACGACTT-3′) (Wakarera et al., 2022), and matrix-assisted laser desorption ionization-time of flight mass spectrometry (MALDI-TOF) (Ultraflextreme, Bruker, Billerica, Massachusetts, USA) adapted from Bagnarino et al., with some modifications. Briefly, each sample was overlaid with 1 uL of matrix solution (saturated *α*-cyano-4-hydroxycinnamic acid in a solvent mixture of 50% acetonitrile, 47.5% Milli-Q water, and 2.5% trifluoroacetic acid) and allowed to air dry completely. Following sample preparation, the target plate was analyzed using a MALDI-TOF mass spectrometer. Spectra were acquired in linear positive-ion mode across a mass-to-charge range of 2,000 to 20,000 Da. The resulting main spectra (MSP) were matched against the Bruker Library. A higher score value indicates greater similarity to a specific reference microorganism within the database (Bagnarino et al., 2022).

**Table 1:** Description of the *Bifidobacterium* cultures used in this study.

No	Isolates in this study	Source	Identification method
1	*B. longum* CICC6186	American Type Culture Collection (ATCC)	/
2	*B. longum* AC15	Human milk	16S sequencing
3	*B. longum* AC16	Human milk	16S sequencing
4	*B. pseudocatenulatum* AC17	Human feces	16S sequencing
5	*B. longum* AC18	Human feces	16S sequencing
6	*B. adolescentis* AC19	Human feces	MALDI-TOF
7	*B. longum* AC20	Human feces	MALDI-TOF

### Growth curve of *Bifidobacterium*

*Bifidobacterium* isolates were cultured, as previously described, for 24 h and then subcultured into fresh medium before monitoring the growth curve. The optical density at OD_600nm_ was monitored every hour for 12 h starting at OD_600nm_ of 0.2 until the cultures reached the stationary phase. The corresponding RCM broth without bacteria was used as a negative control during the observation to control for non-biological change in the medium, such as abiotic turbidity or color change. All the experiments were conducted with three independent biological replicates. The average generation time was calculated using the following equation:


$$n=\frac{\log(\frac{\mathrm{OD}600\mathrm{nm}\_\mathrm{Tn}}{\mathrm{OD}600\mathrm{nm}\_T0})}{\log(2)}$$

- OD_600nm__Tn is the final value of OD_600nm_ at time *n*.
- OD_600nm__T0 is the initial value of OD_600nm_.
- *n* is the number of generations
- The factor of 2 is used because bacteria typically divide by binary fission, where one cell becomes two.

### Determination of tolerance ability towards simulated gastric juice and intestinal juice

*Bifidobacterium* isolates were cultured as described above into the mid-log phase. The culture was centrifuged at 8000 *g* at 4 °C for 10 min, and the pellets were incubated in the Simulated Gastric Juice (SGJ) for 2 h, followed by transferring into the Simulated Intestinal Juice (SIJ) for 3 h. The formulation of SGJ and SIG was adopted from Abdelazez et al. (2022). Isolates were taken out every hour to determine the CFU. An optimal dilution of bacteria culture was spread on RCM plates. All the experiments were conducted with two independent biological replicates. The mean of the log_2_ (CFU) value from the two replicates was used for data visualization.

### Adhesion and competitive adhesion ability of *Bifidobacterium* on colon epithelium cells

A total of 2 × 10^4^ colon epithelium cells (Caco-2 cells) were seeded in 24-well plates and cultured at 37 °C in a 5% CO_2_ incubator for 24 h. Complete media (DMEM+10% FBS) was changed every second day for at least 14 days until a monolayer of Caco-2 cells was formed. *Bifidobacterium* isolates, *S. aureus* ATCC6538, and *E. coli* ATCC8739 were cultured in RCM and TSB broths overnight, respectively. All of them were sub-cultured to mid-log phase at 37 °C. Supernatants were removed after centrifugation and replaced with phosphate-buffered saline (PBS). Bacterial suspensions were standardized to OD_600nm_ of 1.0. Subsequently, 100 μL of each standardized suspension was applied onto Caco-2 monolayers and incubated anaerobically for 2 h. After incubation, Caco-2 cells were washed with PBS three times and lysed with 100 μL of 0.5% Saponin (Cat#sc-280079, Santa Cruz, California, United States) for 15 min. Optimal serial dilution of the cell suspension was spread on RCM or TSB agar plates and incubated at 37 °C. The adhesion assay was conducted with three independent biological replicates. The mean value from the three repeats was calculated and presented in the bar chart.

### Determination of short-chain fatty acids produced by *Bifidobacterium*

The SCFAs analysis method was adapted from Li et al. (2022) with some modifications. *Bifidobacterium* isolates were cultured as described above into mid-log phase. The supernatant of 1 mL bacteria culture was collected by centrifugation and filtered via 0.22 μm membrane. The pH was adjusted to 2–3 by hydrochloric acid before analysis using a column (DB-FFAP 123–3232, Agilent, Santa Clara, USA) on the gas chromatography (GC) with flame ionization detector (FID) (Agilent 7890B, Santa Clara, USA). The oven temperature was set at 80 °C for 2 min, followed by ramping 6 °C per minute until 180 °C, and then held for 2 min. Concentration of different SCFAs, such as acetate, propionate, butyrate, isobutyrate, valerate, and isovalerate, was calculated from standard curves of each SCFA. A standard solution containing a mixture of acetate, propionate, butyrate, isobutyrate, valerate, and isovalerate was prepared, with each constituent at a concentration of 50 mM. This stock was subsequently subjected to a series of two-fold dilutions using Milli-Q water, yielding final concentrations of 25 mM, 12.5 mM, 6.25 mM, 3.125 mM, and 1.5625 mM. The standard curve was constructed by plotting the known concentration of each target analyte against its corresponding mean chromatographic peak area. This test was performed with three biological replicates. The calculated mean concentration of each SCFA among isolates was analyzed using one-way ANOVA.

### Antimicrobial ability of *Bifidobacterium*

The spot-on-law antibacterial assay was adopted from Yadav and Tiwari (2023). Briefly*, Bifidobacterium* isolates were cultured as described above into mid-log phase, and 2 μL of the culture was applied as a single spot on the nutrient agar plates, in which glucose and starch were kept or removed. Semisoft nutrient agar (0.7%) (Cat#CM0110A, Neogen, Lansing, USA) mixed with 10^5^ CFU/mL of overnight cultured pathogens, such as *S. aureus* ATCC6538 and E. coli ATCC8739, including *S. aureus* ATCC6538 and *E. coli* ATCC8739, were overlaid on the RCM plates. Inhibition zones were recorded after 48 h of incubation at 37 °C. All experiments were conducted with three independent biological replicates. The mean inhibition zone of isolates cultured on agar plates with glucose and starch (w-GS) was compared to that of isolates cultured on agar plates without these carbohydrates (w/o-GS) using Student’s *T*-test.

### Isolation of *Staphylococcus aureus*-derived extracellular vesicles

The isolation of SA-EVs was adapted from Jun et al. with some modifications (Jun et al., 2017). Briefly, bacteria were inoculated in TSB broth at 37 °C on an orbital shaker at 250 rpm overnight, followed by sub-culturing into fresh TSB broth until reaching the mid-log phase. After bacterial cells were removed by centrifugation at 8,000 *g* for 20 min at 4 °C, the supernatants were filtered with a 0.22-μm membrane to remove residual bacteria and cellular debris. The filtered supernatant was concentrated with a 100 kDa Amicon centrifugal filter (Cat#UFC910008, Millipore, Germany). The fraction of SA-EVs (>100 KDa fraction) was centrifuged at 150,000 *g* for 3 h at 4 °C, followed by resuspension in PBS. The protein concentration was determined using the BCA assay (Cat#23250, Thermo Fisher Scientific, USA). The isolated SA-EVs were filtered through a 0.22 μm membrane and stored at −80 °C until use.

### Determination of cell viability

Cell viability was assessed by 3-(4,5-dimethylthiazol-2-yl)-2,5-diphenyl tetrazolium bromide (MTT) assay. Briefly, 2×10^4^ cells were seeded per well in a 96-well plate and incubated for 24 h at 37 °C in a humidified atmosphere containing 5% CO_2_. The media was removed, and the cells were treated with varying concentrations of compounds for an additional 24 h. Following treatment, MTT solution (2 mg/mL in PBS) was added to each well, and the plates were incubated at 37 °C for 1 h. The medium was subsequently removed, and 200 μL of DMSO was added to the wells to solubilize the formed formazan crystals. The absorbance was measured on a microplate reader at a wavelength of 540 nm.

### Anti-inflammation capacity of heat-inactivated *Bifidobacterium* on RAW-BLUE™

The supernatant of *Bifidobacterium* isolates at mid-log phase was removed by centrifugation at 8000 *g* for 5 min. The bacterial pellet was washed twice and resuspended in PBS for heat inactivation (HI) at 70 °C for 30 min. The commercially available NF-κB/AP-1 reporter RAW-BLUE™ cell line (Cat#raw-sp) was used to assess the anti-inflammation capacity of HI *Bifidobacterium* isolates. RAW-BLUE™ cell originated from the murine macrophage cell line RAW-264.7 and bore a reporter construct for secreted alkaline phosphatase (SEAP), whose expression is inducible when NF-κB and AP-1 attach to the consensus sequences in the promoter. RAW-BLUE™ cells were cultured in DMEM supplemented with 4.5 g/L glucose, 2 mM L-glutamine, 10% (v/v) heat-inactivated fetal bovine serum, 100 U/mL penicillin, 100 μg/mL streptomycin, 100 μg/mL Normocin™ (Cat#an-tnr-05) or Normocin™ and Zeocin™ (Cat#an-zn-05). RAW-BLUE™ cells were pre-treated with three dosages (10^5^, 10^6^, and 10^7^ CFU/well) of each HI *Bifidobacterium* isolate for 24 h and induced by SA-EVs for 6 h. The supernatant was collected for subsequent analysis. Levels of secreted embryonic alkaline phosphatase, a reporter protein widely used to study promoter activity or gene expression, were monitored by QUANTI-BLUE™ solution (Cat#rep-qbs) and read via a Microplate Reader. The results were normalized to the total protein of the cells. Untreated cells were considered as the negative control, and cells treated with 15 μg of SA-EVs as the positive control. All the aforementioned reagents were purchased from InvivoGen (San Diego, USA). All the experiments were conducted with three independent biological replicates. Normalized average fold change to total protein was shown as mean with standard deviation. Statistical significance was determined by One-way ANOVA.

### The influence of HI *Bifidobacterium* on the polarization of M1 macrophage via NF-κB pathway

Raw 264.7 cells were cultured as previously described, and total RNA was extracted with RNAIso (Cat#9112, TAKARA, Kusatsu, Japan). The extracted RNAs (1,000 ng) were reverse-transcribed into cDNA with PrimeScript™ RT Master Mix (Perfect Real Time) (Cat#RR360A, TAKARA, Kusatsu, Japan) in triplicate, followed by analysis of quantitative PCR (Applied Biosystems QuantStudio 7 Flex Real-Time PCR System, Thermo Fisher, Waltham, USA). The sequences of primers are listed in Table 2. For the western analysis, total protein was extracted by RIPA lysis buffer supplemented with proteinase inhibitor cocktails and phosphorylation inhibitor, respectively. To detect p65 and pp65, 30 μg of cell extracts was resolved via Western blotting, probed with rabbit monoclonal Ab against p65 (Cat#8242) and pp65 (Cat#3033) from Cell Signaling (Danvers, MA, USA), and detected by chemiluminescence (Cat#RM00021P, ABclonal Technology, Woburn, USA). Protein ladder (Cat#26616, Thermo Fisher, Waltham, Massachusetts, USA) was used as a protein marker. The bands obtained were quantified using ImageJ, and the band intensity was normalized to the housekeeping protein (beta-actin). The relative expression of p65 and pp65 was expressed by the values normalized to the negative control. Besides, the level of TNF-α was detected by ELISA (Cat#RP01071, ABclonal Technology, Woburn, USA). All experiments were conducted with three independent biological replicates. Results were expressed as the mean with the standard deviation of these replicates. Statistical analysis was performed using one-way ANOVA.

**Table 2:** Primer sequence for qPCR analysis.

Target	For 5′-3′	rev 5′-3′	Reference
CD80	TGCTGCTGATTCGT CTTTCAC	GAGGAGAGTTGTAACGGCAAG	Taddio et al. (2021)
TNFA	AGCCCACGTCGTAGCAAAC	TTTGAGATCCATGCCGTTGG	Zhu et al. (2022)
CD206	AAACACAGACTGACCCTTCCC	GTTAGTGTACCGCACCCTCC	Li et al. (2024)
Arg1	CTCCAAGCCAAAGTCCTTAGAG	GGAGCTGTCATTAGGGACATCA	Li et al. (2024)
GAPDH	GCACCGTCAAGGCTGAGAAC	TGGTGAAGACGCCAGTGGA	Li et al. (2024)

## Results

### Growth curve of *Bifidobacterium* in the RCM broth

**Figure 1:**
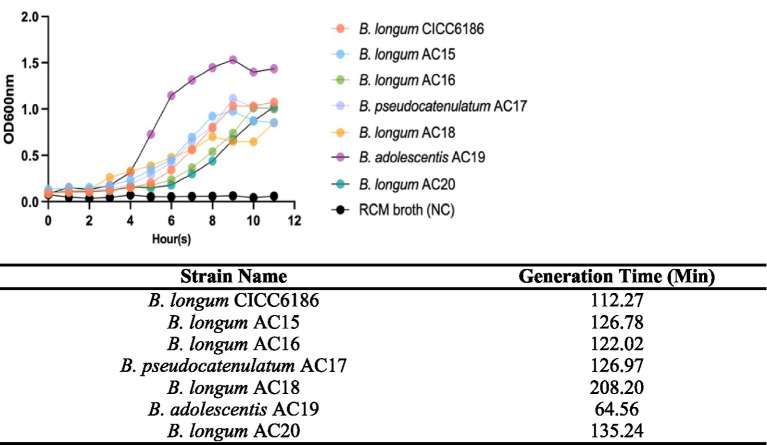
Growth curve and generation time of seven *Bifidobacterium* isolates cultured in RCM broth. Each point was done in triplicate, and data were shown as the average value.

Those *Bifidobacterium* isolates were recovered exclusively on RCM agar, suggesting that this agar offers the optimal nutritional composition for their cultivation. Therefore, RCM broth was selected for all subsequent growth curve experiments. As shown in Figure 1, the growth rate of *B. adolescentis* AC19 in RCM broth was the highest, with a generation time of 64.56 min among seven *Bifidobacterium* isolates under optimal conditions. On the contrary, *B. longum* AC18 has the longest doubling time of 208.20 min among all. The rest of the *Bifidobacterium* isolates, *B. longum* CICC6186, *B. longum* AC15, AC16, and AC20, and *B. pseudocatenulatum* AC17, had similar generation times, ranging from 112.27 to 135.24 min.

### *Bifidobacterium* isolates exhibited gastric juice tolerance but were susceptible to intestinal juice

**Figure 2:**
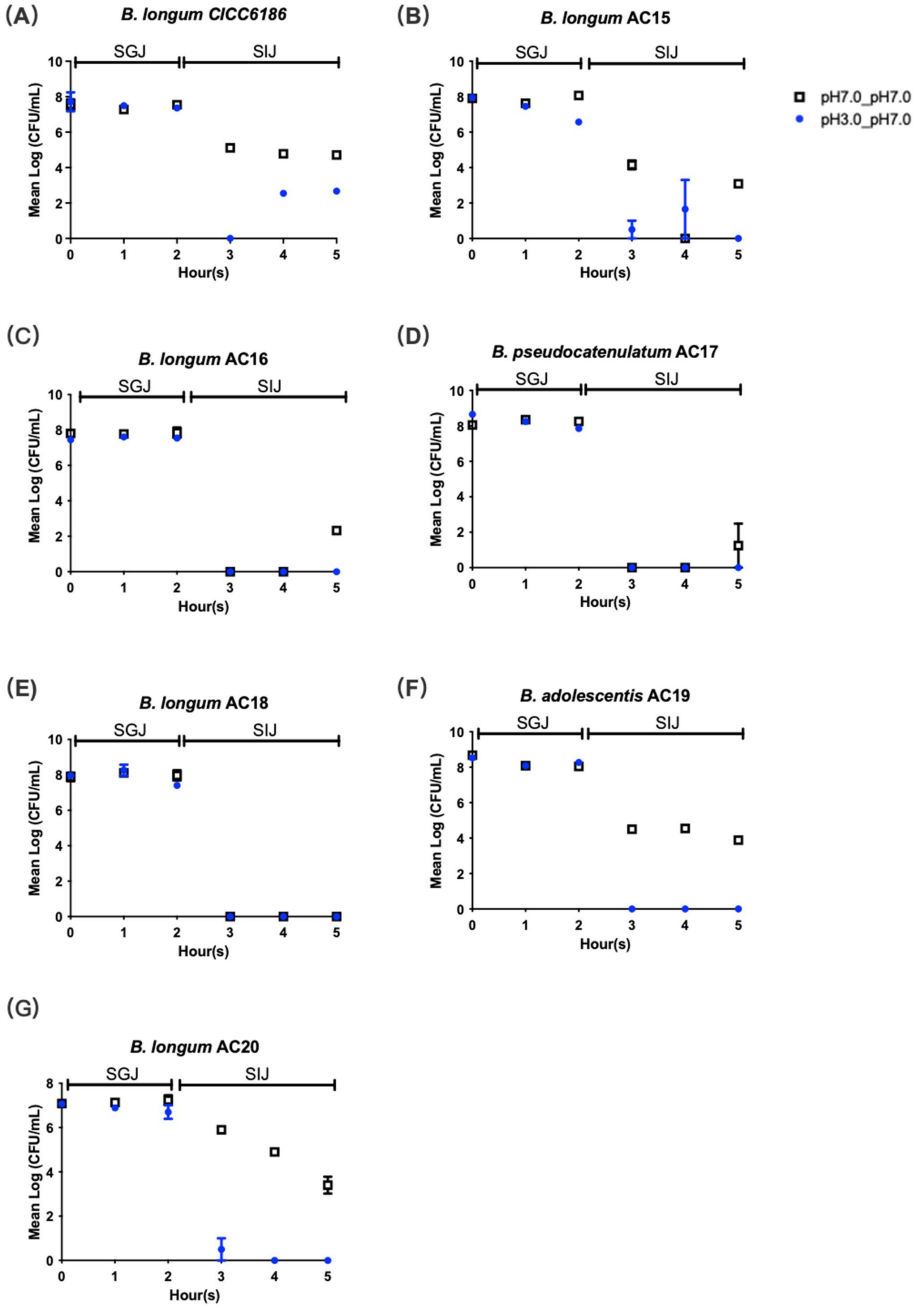
Tolerance of seven *Bifidobacterium* isolates. *B. longum* CICC6186 **(A)**, *B. longum* AC15 **(B)**, *B. longum* AC16 **(C)**, *B. psedocatenulatum* AC17 **(D)**, *B. longum* AC18 **(E)**, *B. adolescentis* AC19 **(F)**, *B. longum* AC20 **(G)** toward simulated gastric juice (SGJ) and simulated intestinal juice (SIJ). Blue dots represent bacteria incubated at pH 3.0 for 2 h followed by pH 7.0 for 3 h. Black squares represent bacteria incubated at a constant pH 7.0 as the control. Each point was done in duplicate, and the data were shown as mean ± SEM values.

The tolerance to gastric juice and intestinal juice is crucial for probiotics, as it allows them to survive and thrive in the harsh environment of the human GI tract. *Bifidobacterium* isolates have varying levels of tolerance to simulated GI conditions. The seven *Bifidobacterium* isolates withstood the challenge of simulated gastric juice for 2 h, while some of them struggled to survive in the simulated intestinal juice for 3 h, with the log (CFU/mL) number almost reaching zero (Figure 2). *B. longum* CICC6186 and *B. longum* AC15 demonstrated modest resistance to SIJ, as viable bacteria remained detectable throughout the incubation period, yet remaining at very low levels, with the mean log (CFU/mL) values of 2.66 ± 0.08 and 2.47 ± 1.65, respectively. The rest of the isolates cannot stand the challenge of SIJ for 1 h with the log (CFU/mL) number equal to zero.

### *Bifidobacterium* isolates adhered to and competitively excluded *S. aureus* and *E. coli* from Caco-2 cells

**Figure 3:**
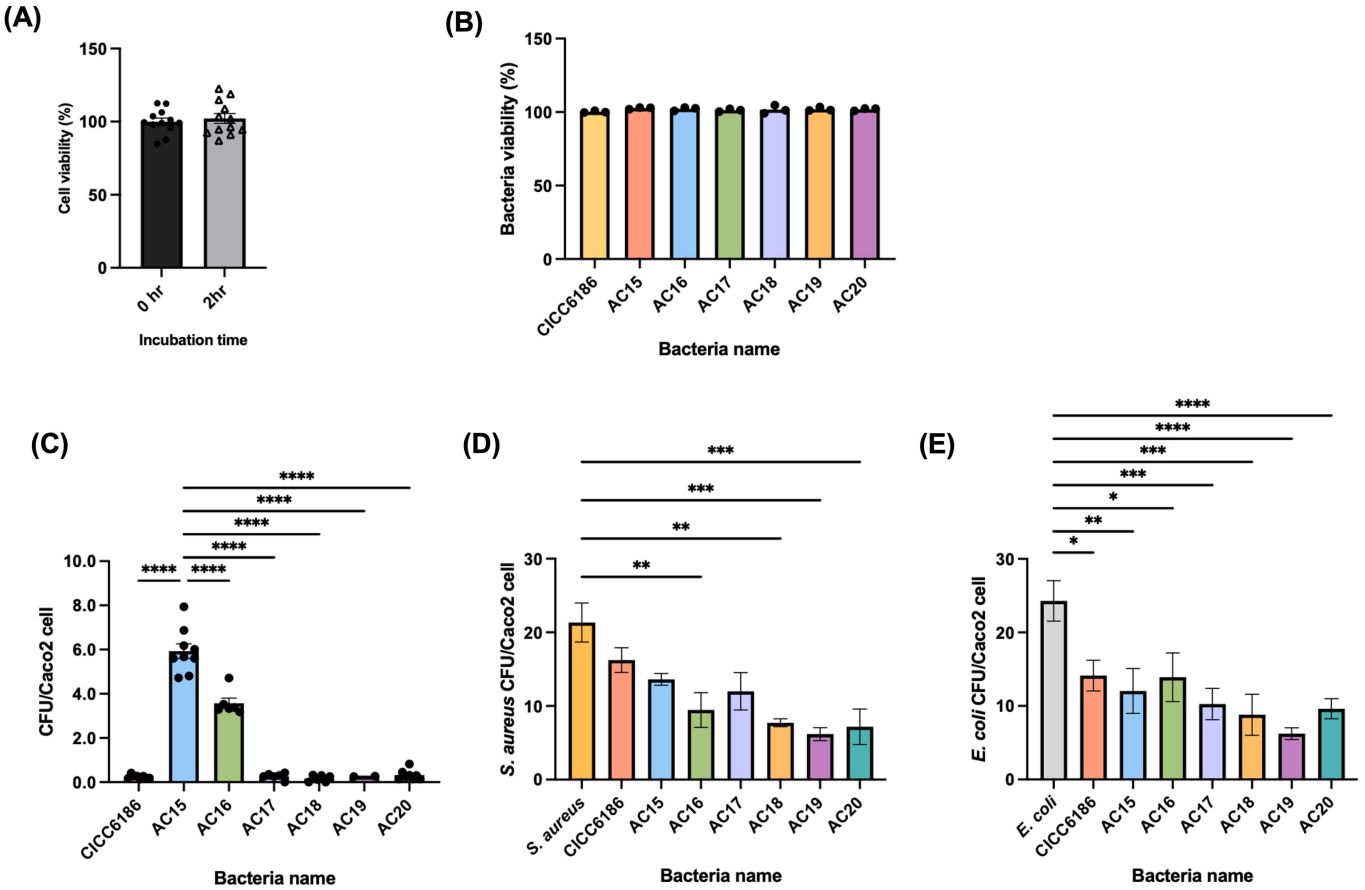
Adhesion and competitive adhesion ability of *Bifidobacterium* isolates on Caco-2 cells. Viability of Caco-2 cells under anaerobic conditions for 2 h **(A)**. Viability of *Bifidobacterium* isolates in lysis buffer (0.5% Saponin) **(B)**. Adhesion ability of *Bifidobacterium* isolates cultured in RCM broth Caco-2 **(C)**, and competitive adhesion ability of *Bifidobacterium* isolates against *S. aureus* **(D)** and *E. coli* **(E)**. Each point was repeated in triplicate, and the data were shown as mean ± SEM values. All groups were compared to *S. aureus* and *E. coli*. One-way ANOVA, ****p < 0.0001, ***p < 0.001, **p < 0.01, *p < 0.05.

As the adhesion and competitive adhesion assays were conducted under anaerobic conditions, the viability of Caco-2 cells under this environment was assessed. The results showed that Caco-2 cells could survive anaerobic conditions for up to 2 h (Figure 3A). Additionally, since the assay requires lysing Caco-2 cells with lysis buffer (0.5% saponin), the effect of this buffer on the viability of the *Bifidobacterium* strains was also evaluated. Seven *Bifidobacterium* strains were found to be tolerant to 0.5% saponin under the tested conditions (Figure 3B). The adhesion and competitive adhesion abilities to human epithelial cells of the *Bifidobacterium* isolates were tested. Those abilities are crucial for their probiotic functionality and potential health benefits by preventing the invasion and colonization of gut pathogens. As shown in Figure 3C, *B. longum* AC15 and AC16, isolated from human milk, had the outstanding adhesion ability of all, with 6.0 ± 1.1 and 3.6 ± 0.6 bacteria per Caco-2 cell, respectively, while the rest of *Bifidobacterium* isolates barely adhered to the Caco-2 cells (less than one bacterium/Caco-2 cell). The competitive adhesion properties against *S. aureus* and *E. coli* amongst the seven *Bifidobacterium* isolates varied. *B. adolescentis* AC19 showed the distinguished ability to competitively exclude the colonization of both Gram-positive *S. aureus* and Gram-negative *E. coli* from Caco-2 cells. It successfully reduced the number of *S. aureus* and *E. coli* per Caco-2 cell from 21.3 ± 4.6 and 24.2 ± 5.5 to 6.2 ± 1.5 and 6.2 ± 2.4, respectively (Figures 3D,E). The competitive adhesion ability of *B. longum* AC15 is higher in *E. coli* (50.4% reduction) than *S. aureus* (36.2% reduction) on Caco-2 cells.

### *Bifidobacterium* fermentation yielded acetate as the predominant detectable SCFAs

**Figure 4:**
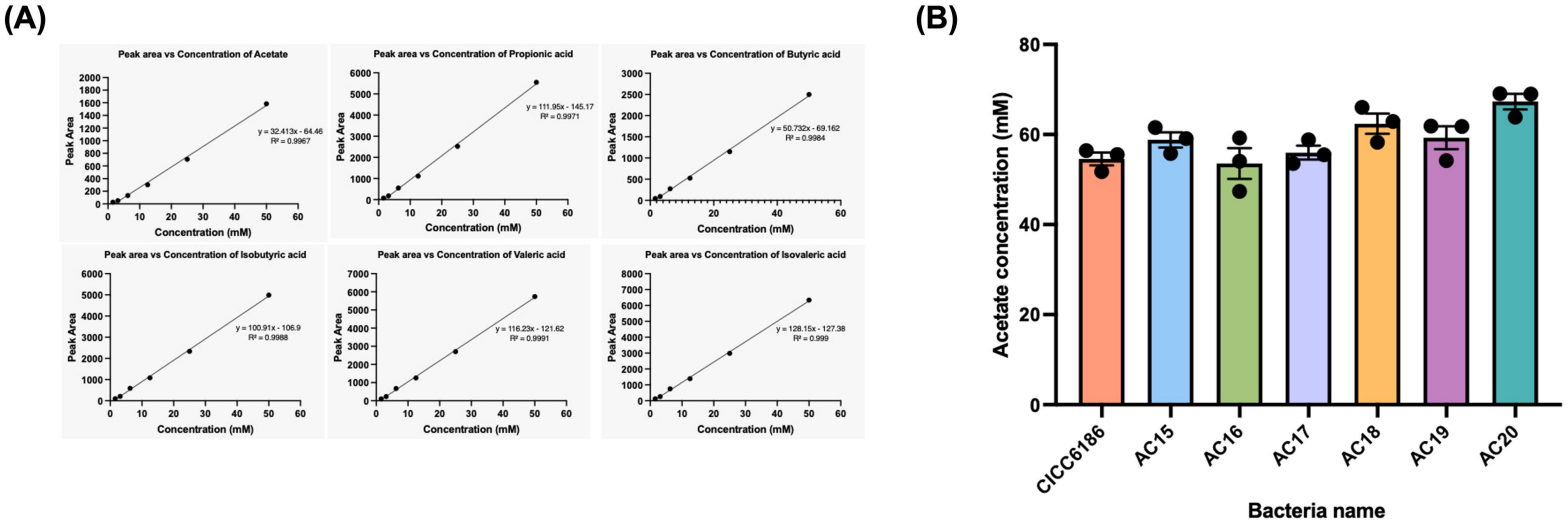
Comparison SCFAs production ability among seven *Bifidobacterium* isolates. Standard curve of six SCFAs, acetate, propionic acid, butyric acid, isobutyric acid, valeric acid, and isovaleric acid **(A)**. Comparison of the amount of acetate produced by seven *Bifidobacterium* isolates **(B)**. Each point was repeated in triplicate, and the data were shown as mean ± SEM values. One-way ANOVA, ****p < 0.0001, ***p < 0.001, **p < 0.01, *p < 0.05.

SCFAs are the fermentation products of dietary fibers by certain gut microbiota, which have a variety of impacts on host metabolism and can be utilized as an energy source supporting the intestinal barrier system. *Bifidobacterium*, as the GI tract’s ubiquitous inhabitant, serves to increase the production of SCFAs in the gut and regulate host energy metabolism. The six SCFAs produced by *Bifidobacterium* were analysed via GC-FID (Figure 4B). Standard curves for the six SCFAs were established and are presented in Figure 4A, demonstrating linear relationship between concentrations and peak areas across the investigated ranges. The acetate production ranged from 53.56 mM to 67.31 mM, with no statistical differences among the tested isolates. The rest of the five SCFAs were below the detection limit (data not shown).

### *Bifidobacterium* isolates inhibited the growth of *S. aureus* and *E. coli*

As shown in Table 3, all tested *Bifidobacterium* isolates displayed different levels of inhibition zone against the two typical Gram-positive and Gram-negative pathogens, with the inhibition radius ranging from 0.53 cm to 0.90 cm and 0.43 cm to 1.07 cm against *S. aureus* ATCC6538 and *E. coli* ATCC8739, respectively, in the presence of carbohydrates. Amongst all analyzed *Bifidobacterium*, *B. longum* AC20 had the biggest inhibitory zone against *S. aureus,* with a value of 0.86 ± 0.15 cm, while *B. longum* CICC6186 was the best one against *E. coli* with the inhibition radius of 1.07 ± 0.12 cm. Moreover, *Bifidobacterium* strains AC16 and AC19 showed a significantly wider inhibition zone against *S. aureus* when they were cultured in the RCM plates with carbon sources (1 g/L starch and 5 g/L glucose) than in the absence of carbon sources, suggesting that acid production is one of the major factors contributing to their antimicrobial ability. In terms of inhibition ability toward *E. coli*, all the *Bifidobacterium* isolates presented distinct inhibition ability in RCM plates with carbon sources. Interestingly, all *Bifidobacterium* isolates showed inhibition zones against both pathogens in the absence of carbon sources, implying that they produced other antimicrobial substances other than acid, such as bacteriocins.

**Table 3:** Inhibitory effects of *Bifidobacterium* isolates toward *S. aureus* ATCC6538 and *E. coli* ATCC8739 of *Bifidobacterium* isolates.

Strain name	Against *S. aureus* ATCC6538		Against *E. coli* ATCC8739	
	w/o-GS (cm)	w-GS (cm)	w/o-GS (cm)	w-GS (cm)
*B. longum* CICC6186	0.57 ± 0.06	0.77 ± 0.12	0.47 ± 0.06***	1.07 ± 0.12
*B. longum* AC15	0.43 ± 0.06	0.53 ± 0.06	0.27 ± 0.06***	0.63 ± 0.06
*B. longum* AC16	0.47 ± 0.06**	0.80 ± 0.10	0.53 ± 0.06*	0.67 ± 0.06
*B. longum* AC17	0.53 ± 0.06*	0.66 ± 0.06	0.30 ± 0.01*	0.43 ± 0.06
*B. pseudocatenulatum* AC18	0.70 ± 0.01*	0.90 ± 0.10	0.27 ± 0.06*	0.43 ± 0.06
*B. adolescentis* AC19	0.43 ± 0.06**	0.80 ± 0.10	0.23 ± 0.06*	0.43 ± 0.06
*B. longum* AC20	0.66 ± 0.06	0.86 ± 0.15	0.30 ± 0.01***	0.77 ± 0.06

Experiments were conducted in triplicate, and data are expressed as the mean ± SEM. The diameter of the inhibition zone produced by each *Bifidobacterium* isolate was compared between RCM agar plates prepared without (w/o-GS) and with (w-GS) the addition of glucose and starch. Student *T*-test, ****p < 0.0001, ***p < 0.001, **p < 0.01, *p < 0.05.

### HI *Bifidobacterium* attenuated inflammatory response in macrophage

**Figure 5:**
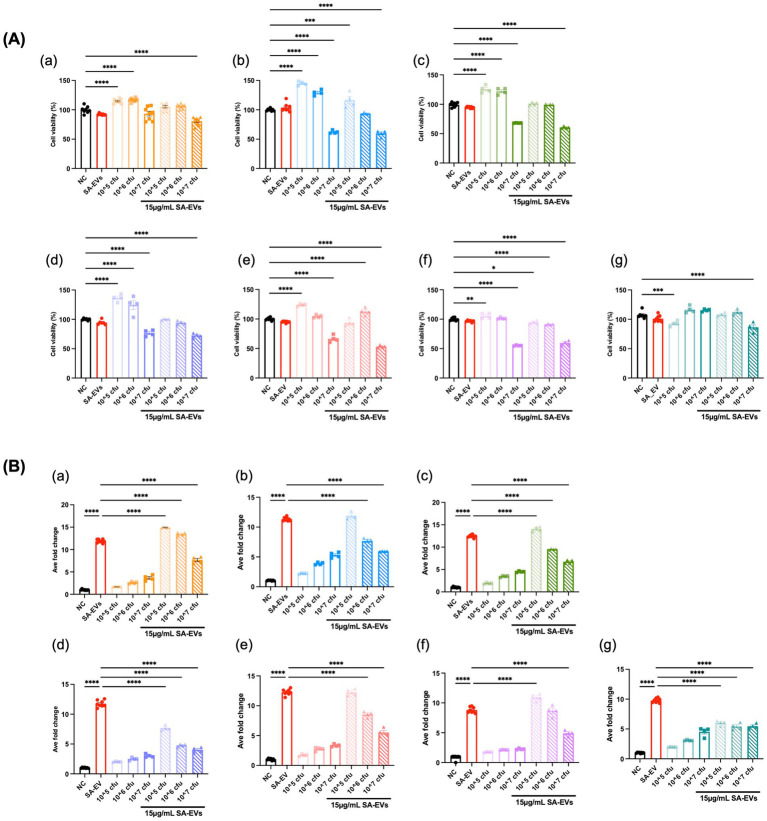
Effects of HI *Bifidobacterium* on the viability and anti-inflammatory cytokine expression of RAW-BLUE™ macrophages. RAW-BLUE™ cells were treated with HI *Bifidobacterium* isolates in the presence of SA-EVs. Cell viability was assessed using the MTT assay **(A)**. The fold change in SEAP activity was measured to assess the modulation of the inflammatory response via the NF-κB/AP-1 pathway **(B)**. The isolates tested were: *B. longum* CICC6186 (a), *B. longum* AC15 (b), *B. longum* AC16 (c), *B. longum* AC17 (d), *B. pseudocatenulatum* AC18 (e), *B. adolescentis* AC19 (f), and *B. longum* AC20 (g). All assays were performed in triplicate, and data are presented as mean ± SEM. All groups compared with *SA-EVs. One-way ANOVA, ****p < 0.0001, ***p < 0.001, **p < 0.01, *p < 0.05.

Most of the *Bifidobacterium* strains were non-viable after passage through the intestine. Furthermore, the incompatible culture conditions required for viable *Bifidobacterium* (strict anaerobic environment) and macrophages (aerobic environment) necessitated the use of heat-inactivated bacteria for the co-culture experiments. The HI *Bifidobacterium* isolates were analyzed for their anti-inflammatory capacity on the reporter cell line, RAW-BLUE™ cell line. The MTT assay was used to monitor the viability of RAW-BLUE™ cells. As shown in Figure 5A, the treatment of the lowest and the middle dosages of HI *Bifidobacterium* (10^5^ CFU and 10^6^ CFU/well) alone may not cause significant cell death. On the contrary, some *Bifidobacterium* isolates of those two dosages promoted the growth of RAW-BLUE™ cells. The highest dosage (10^7^ CFU/well) of several HI *Bifidobacterium* isolates reduced the cell viability to less than 60%. Taken together, those results indicated that 10^5^ and 10^6^ CFU/well were the suitable dosages for this experiment. All the *Bifidobacterium* isolates tested in this experiment showed great anti-inflammatory effects against SA-EVs at the dosage of 10^6^ CFU/mL. *B. longum* AC17 and AC20 are the two most potent strains having anti-inflammatory capabilities amongst seven *Bifidobacterium* isolates since they began to exhibit considerable anti-inflammatory effects at the lowest dosage of 10^5^ CFU/mL. The remaining *Bifidobacterium* isolates only began to show significant positive effects in the middle dosage of 10^6^ CFU/mL (Figure 5B).

### Anti-inflammatory effects of HI *Bifidobacterium* via the suppression of M1 macrophage polarization

**Figure 6:**
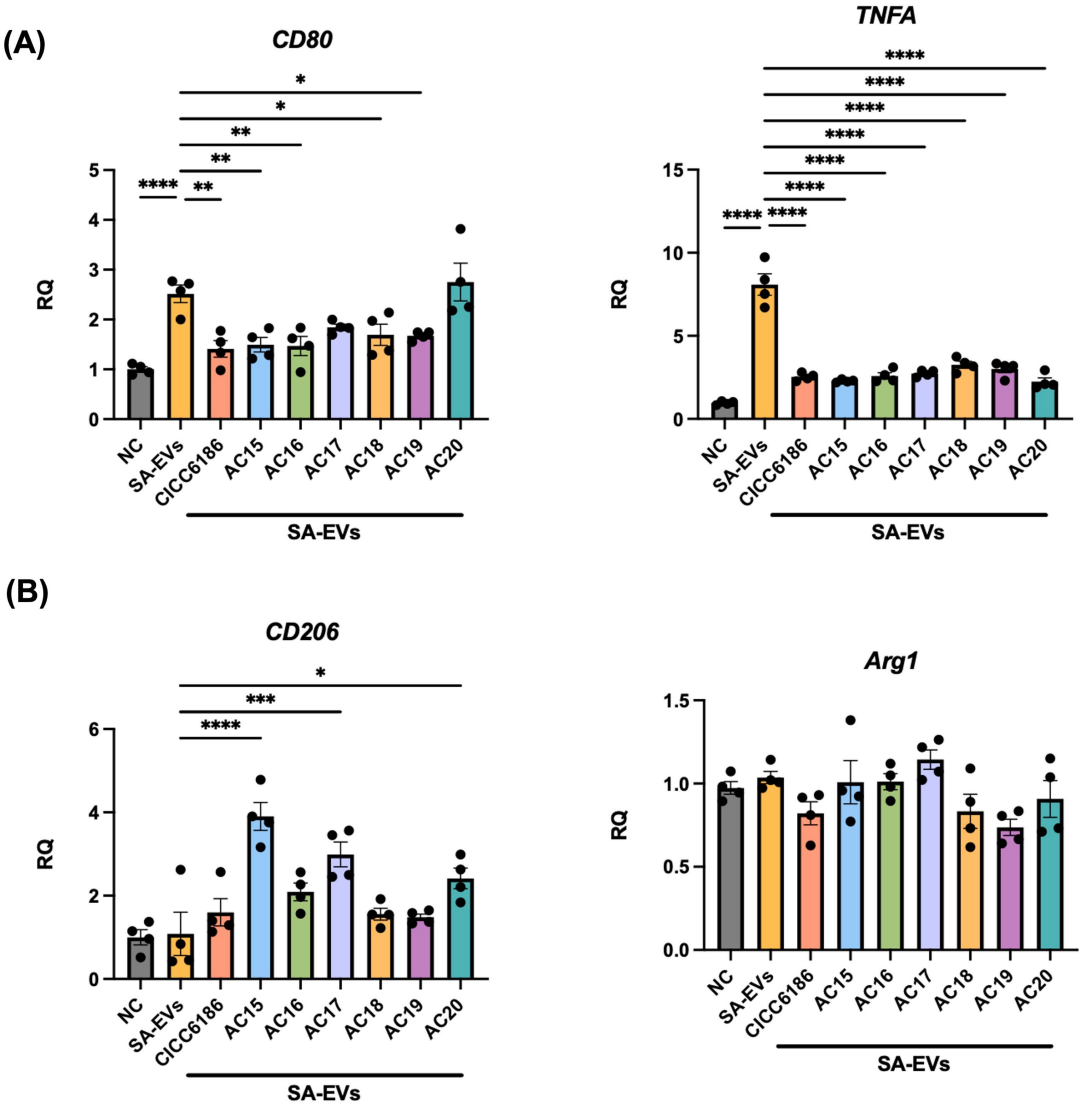
The mRNA expression of macrophage polarization-associated genes in Raw 264.7 cells. The mRNA levels of M1 markers **(A)** and M2 markers **(B)** were analyzed and compared between the SA-EVs and seven *Bifidobacterium* isolates. Each point was repeated in triplicate, and the data were shown as mean ± SEM values. All groups were compared to *SA-EVs group. One-way ANOVA, ****p < 0.0001, ***p < 0.001, **p < 0.01, *p < 0.05.

The expression levels of M1 macrophage polarization-related genes, *CD80* and *TNFA*, were significantly upregulated by SA-EVs, while pretreatment with all tested HI *Bifidobacterium* isolates, except *B. longum* AC17 and AC20, could prevent this polarization. The expression of M2 macrophage polarization-related genes *CD206* and *Arg* was not activated by SA-EVs. However, one marker of M2 macrophage, *CD206*, was observed to be higher in *B. longum* AC15, AC17, and AC20 than in the rest of the groups with statistical significance. The M2 marker, *Arg1*, showed no significant change amongst all groups (see Figure 6).

**Figure 7:**
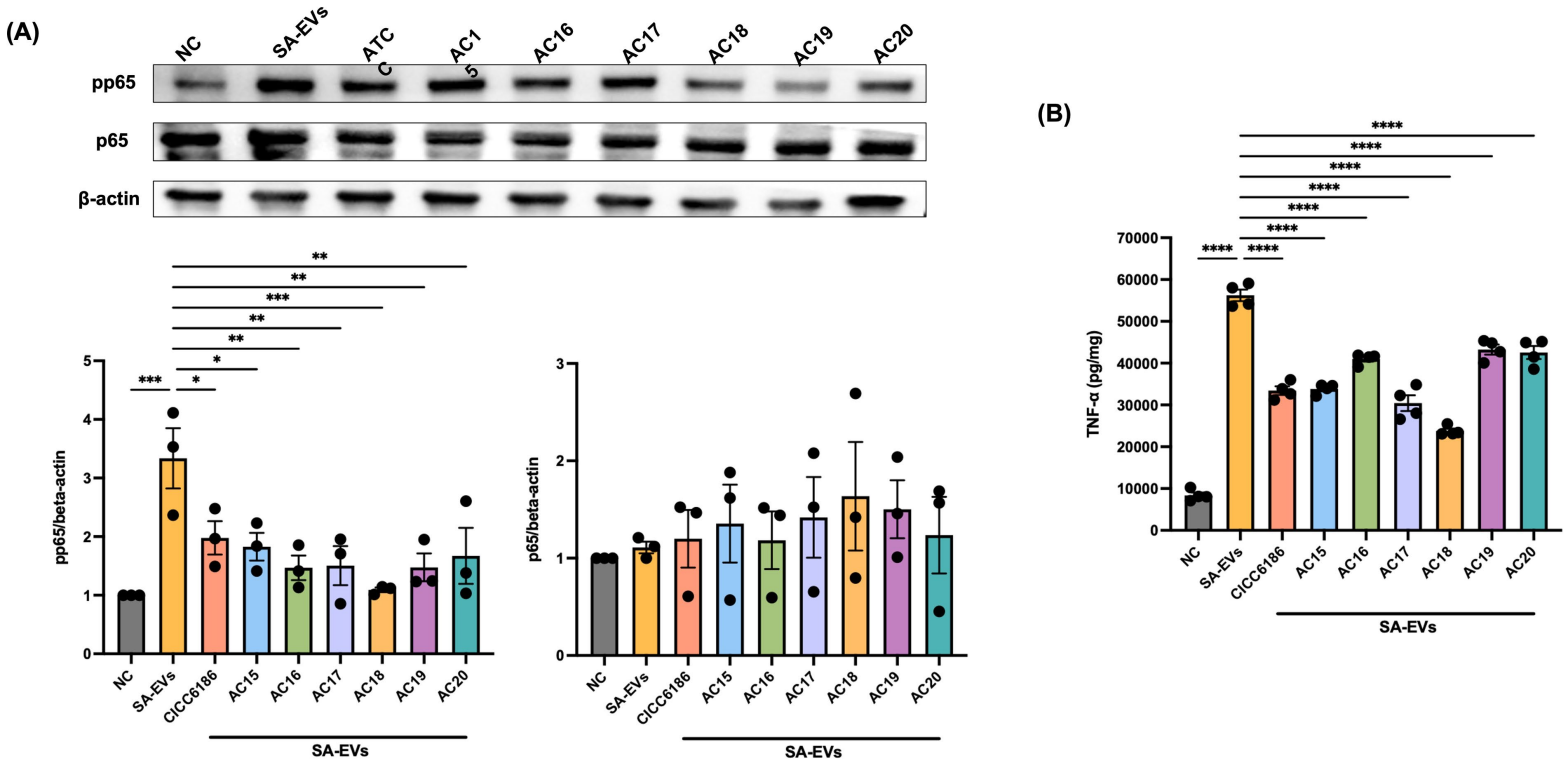
Determination of nuclear translocation of the NF-κB subunit by western blot **(A)** and TNF-α activation by ELISA **(B)**. Each point was repeated in triplicate, and the data were shown as mean ± SEM values. All groups were compared to *SA-EVs group. One-way ANOVA, ****p < 0.0001, ***p < 0.001, **p < 0.01, *p < 0.05.

To further confirm which pathway was influenced by HI *Bifidobacterium* isolates to prevent the inflammation induced by SA-EVs on Raw 264.7 cells, TNF-*α*, as the transcription factor p65, and its phosphorylated form (pp65) were analyzed via ELISA and western blot, respectively, with beta-actin as the reference protein. Induction with SA-EVs alone on Raw 264.7 cells activated the phosphorylates of the NK-κB signaling pathway, while pretreatment with those seven HI *Bifidobacterium* isolates could prevent this occurrence. Besides, there was a notable decrease in the level of NF-κB-dependent protein, TNF-α, in those seven treatment groups (see Figure 7).

## Discussion

For obtaining *Bifidobacterium* via either human milk or daily supplements, the first challenge is passing through the gastric digestion, where the bacteria are exposed to low pH (3.0 in an empty stomach) and high pepsin concentration in the gastric juice. The two stresses have the potential to be bacteriostatic or bactericidal. The second challenge is the natural barrier, the duodenal loop of the small intestine, where the majority of bile salt exposure happens. This procedure could destroy the lipids in the cell membrane, injuring cell permeability and inhibiting many bacteria (Azer et al., 2017). To exert beneficial effects on the human body, surviving the harsh environment of the stomach and GI tract is critical for probiotics. In our study, selected *Bifidobacterium* isolates remained viable under the low pH environment of SGJ, while almost none of them could survive after incubating with 3.0 g/L of bile salt for 3 h. This observation is consistent with previous findings (Wendel, 2021). Those strains were vulnerable to bile salt, probably due to a lack of bile salt hydrolase, which helped to break down bile salts into free amino acids and bile acids (Kang et al., 2025). Certain strains may harbor bile salt hydrolases, which can generate deconjugated bile salts during biotransformation and protect them from the harm of bile salt, thereby surviving in the small intestine (Bustos et al., 2012). In this study, *B. longum* AC15 and *B. longum* CICC6186 appeared to harbor bile salt hydrolase as they survived under the challenge of SIJ. Genetic analysis was not conducted, as our primary goal was to screen for strains capable of enduring this challenge.

One of the classical selection criteria for probiotic bacteria is adhesion and competitive adhesion ability. For isolates that could withstand the harsh conditions in the upper GI tract, the subsequent primary objective is to colonize the intestinal epithelium. In this study, two isolates from human milk (*B. longum* AC15 and AC16) displayed superior adhesion ability. The adhesion of bifidobacteria to the host is a multifactorial process involving cell surface structure, genetic determinants, and receptor specificity, etc (Gorreja and Walker, 2022). A key structural feature is the expression of sortase-like pili protein, which mediates direct binding to host proteins such as fibronectin and mucin (Westermann et al., 2016). Furthermore, surface polymers like exopolysaccharides (EPS) and lipoteichoic acids (LTA) of certain bifidobacteria also facilitate the non-specific hydrophobic interactions with gut mucosa, thereby promoting their stable colonization (Castro-Bravo et al., 2018). However, the specific reasons responsible for the good adhesion ability of these two isolates remain to be uncovered. Another critical health benefit of bifidobacteria is resistance against colonization and infection of pathogens (Monteagudo-Mera et al., 2019; Gorreja and Walker, 2022). In this study, *B. adolescentis* AC19 and *B. longum* AC20 exhibited the best competitive bacteria, excluding 71.0% to 60.37% of *S. aureus* ATCC6538 and 74.3% to 66.38% of *E. coli* ATCC8739 that bound to Caco-2 cells, respectively. This finding is consistent with previous reports showing that most *Bifidobacterium* strains achieved at least 70% exclusion of both *S. aureus* and *E. coli* (Ronkainen et al., 2024). This exceptional competitive exclusion may be mediated by several synergistic mechanisms, most notably direct receptor competition and pathogen co-aggregation. These primary actions are further augmented by competitive nutrient uptake and the production of antimicrobial compounds, such as SCFAs (Zawistowska-Rojek et al., 2022).

The capacity of probiotics to produce SCFAs is increasingly recognized as a critical functional attribute influencing strain selection and application strategy (Nami et al., 2025). Their functional profile not only reflects metabolic activity but also demonstrates potential health outcomes in the host. For instance, acetate and propionate are more closely associated with systemic metabolic modulation and pathogen inhibition (Hosmer et al., 2024). Butyrate could provide the energy to colonocytes, protect the gut barrier integrity, and have anti-inflammatory effects (Korsten et al., 2023). From our results, six isolates produced comparable amounts of SCFAs, with acetate being the most abundant, which ranged between 53.56 and 67.31 mM, and the rest were below the limit of detection. The observed antimicrobial activity of *Bifidobacterium* may be mechanistically linked to their SCFAs output. The evaluation of SCFAs enables a more rationale-driven probiotic selection, guiding whether a strain is suited for gut health maintenance, metabolic health applications, or targeted antimicrobial use.

All assessed isolates produced pronounced inhibition zones against the representative pathogens, with diameters ranging from 0.53 to 0.90 cm against *S. aureus* and from 0.43 to 1.07 cm against *E. coli*. These findings were consistent with previous reports, which documented inhibition zones ranging from 0.638 to 1.485 cm against *S. aureus* and from 0.625 to 1.285 cm against *E. coli* (Tham et al., 2012; Ji et al., 2025). The spot-on-lawn assay employed in this study was selected for its exceptional speed and technical simplicity, as it requires minimal equipment and preparation by directly spotting the test compound onto pre-inoculated agar plate, bypassing steps like well cutting or disk placement (Kobras et al., 2023). Acid production is considered a major reason for the antimicrobial ability of bacteria. However, in the agar plate without the carbon source, the level of inhibition zone of *B. longum* CICC6186 and *B. longum* AC15 against *S. aureus* was not significantly influenced. These results suggested another highly possible mechanism, namely bacteriocins, which are known to harbor a broad activity spectrum toward both Gram-positive bacteria, e.g., *Streptococcus*, *Staphylococcus* and *Clostridium*, and Gram-negative bacteria, such as *Salmonella*, *Shigella*, and *E. coli* (Moroni et al., 2006; Cheikhyoussef et al., 2009). However, this study was limited by the lack of direct quantification of bacteriocin production. Another recognized limitation of the spot-on-lawn method is its comparatively reduced precision and reproducibility relative to standardized diffusion techniques.

The anti-inflammatory effects of HI *Bifidobacterium* isolates exhibited significant variation across different species. Based on our previous SIJ tolerance results, those isolates were sensitive to intestinal conditions. Consequently, their survival rate is expected to be low upon transit through the GI tract. Given this limitation, the present study was designed to investigate whether the inactivated *Bifidobacterium* retains anti-inflammatory properties. Besides, the incompatible culture conditions of viable *Bifidobacterium* (strict anaerobic condition) and macrophages (aerobic condition) further make it necessary. As a result, the heat-inactivated form of *Bifidobacterium* isolates was applied on RAW-BLUE™ cell line, which provides a high-throughput platform for (PRR) stimulation. However, a key limitation of using inactivated bacteria is their inability to replicate, metabolize, or interact dynamically with the host. Consequently, they cannot accurately model the complex, live in a microbial ecosystem within the gastrointestinal tract, where metabolic activity and population dynamics are central to function. SA-EVs were used to induce inflammation in this study, as it highly correlates with many inflammatory diseases, such as atopic dermatitis (Kim et al., 2012, 2018). As demonstrated in our results, SA-EVs effectively induced the inflammation and the polarization of macrophages from the M0 to the M1, with a significant upregulation of the key polarization markers. The phenotypic shift of naïve macrophages toward M1 was verified by the overexpression of *CD80*, a well-known surface marker of M1 macrophages (Oliveira et al., 2019). Concurrently, the significant elevation in *TNFA* expression was induced, signifying a pro-inflammatory state of M1 macrophage (Song et al., 2023). Pretreatment with the HI *Bifidobacterium* isolates could effectively reduce the inflammatory response. All tested isolates demonstrated pronounced anti-inflammatory activity at a dosage of 10^6^ CFU, whereas only a few showed notable effects at the lower dosage of 10^5^ CFU. Besides, they prevented the polarization and attenuated the pro-inflammatory states, especially *B. longum* AC17 and *B. longum* AC20. This protective effect was proven by the reduced expression of *CD80* and *TNFA*, which correlated with the suppression of macrophage inflammatory activity. The underlying mechanism likely involves the inhibition of p65 phosphorylation, thereby blocking the downstream activation of the NF-κB signaling pathway and further preventing the development of inflammation. This finding is consistent with numerous studies reporting that various *Bifidobacterium* strains exert potent anti-inflammatory effects on macrophages (Hao et al., 2025). Furthermore, these strains have been shown to inhibit M1 polarization while promoting M2 polarization of macrophages (Fontana et al., 2021). This study conducted a small-scale screening to identify potential *Bifidobacterium* candidates with anti-inflammatory activity against SA-EVs. The number of bacterial strains tested was limited by sample size and source availability. Future studies should include participants across a wider age range to increase the diversity of *Bifidobacterium* isolates. Subsequently, the most promising isolates will be evaluated in relevant animal models of SA-EVs-triggered inflammation. Furthermore, this study focused solely on the health-associated functional characteristics of *Bifidobacterium* and did not investigate the underlying mechanisms. Additional research is needed to elucidate these mechanisms.

## Conclusion

In summary, assessment of the selected *Bifidobacterium* isolates reveals that each strain possesses unique characteristics. This study aimed to identify the most potent strains for attenuating inflammation induced by SA-EVs, a key driver of atopic dermatitis. Among all the tested isolates, *B. longum* AC15 displayed a relatively promising profile, demonstrating a strong SGJ and SIJ tolerance, as well as abilities in adhesion and competitive adhesion toward pathogens on enterocytes. Although its SCFAs production and anti-inflammatory effects were comparable to those of other strains, this combination of resilience and adhesion highlights its potential for further investigation in the context of SA-EVs-associated inflammation. To our knowledge, this is the first study to screen *Bifidobacterium* strains specifically for their ability to counteract SA-EV-induced inflammatory responses, underscoring the importance of strain-specific selection for targeted therapeutic applications. Current limitations, including the exclusive use of *in vitro* models and the absence of supporting genomic analysis, define clear objectives for future research.

## Data Availability

The original contributions presented in the study are included in the article/Supplementary material, further inquiries can be directed to the corresponding author.
