# Supplementary material for: Elucidation of Bifidobacterium isolates from human milk and feces: investigating their anti-inflammatory effects on raw 264.7 via NF-κB signaling pathway
Source: Front Microbiol. 2026 Mar 16;17:1763675. doi: 10.3389/fmicb.2026.1763675 (PMC13033738; doi:10.3389/fmicb.2026.1763675)
Supplement: Supplementary file 1 [file Data_Sheet_1.PDF]

Supplementary Material

Supplement figures:

Membrane 1_p65+beta actin

Chemi

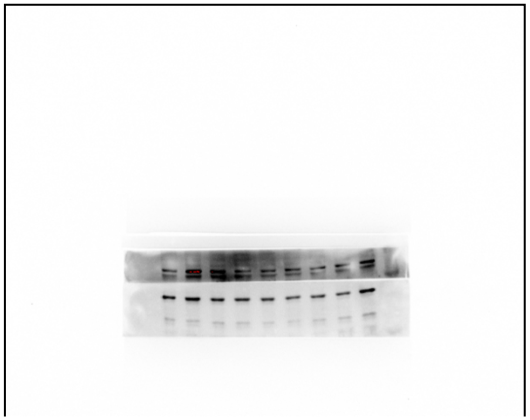

Membrane

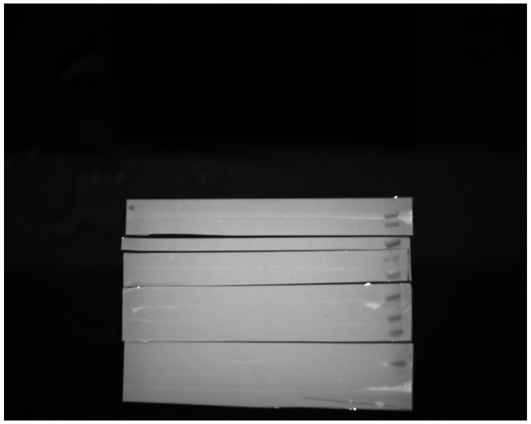

Overlay

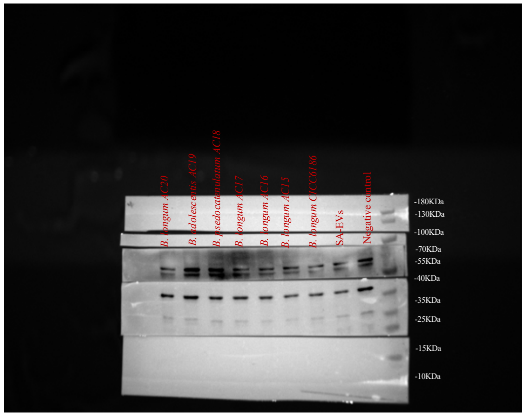

Membrane 1_pp65-1

Chemi

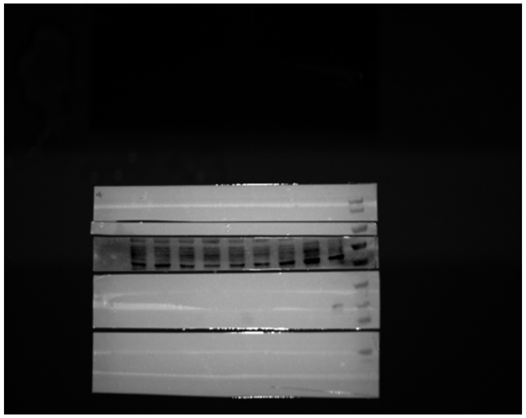

Membrane

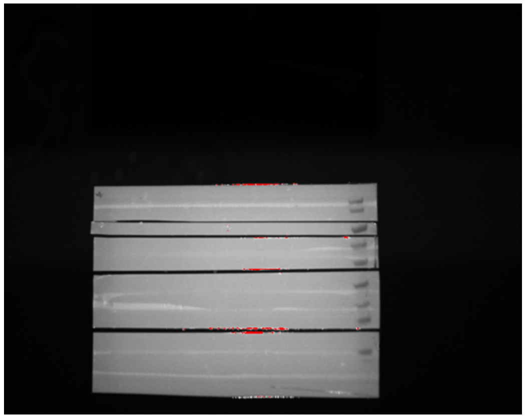

Overlay

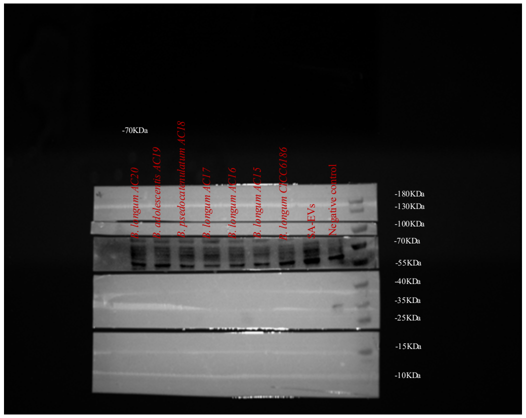

Membrane 1_p65-1

Chemi

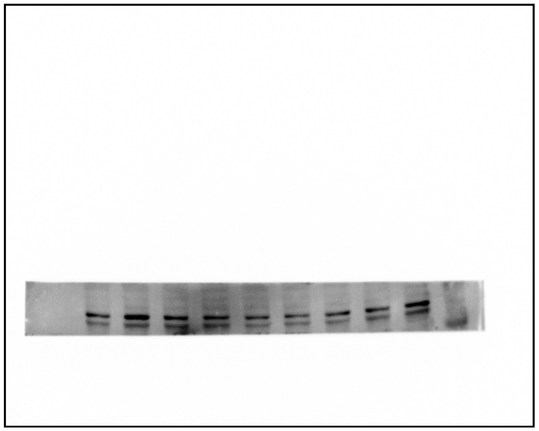

Membrane

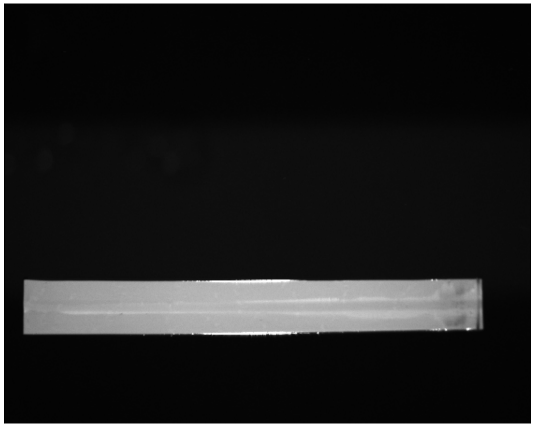

Overlay

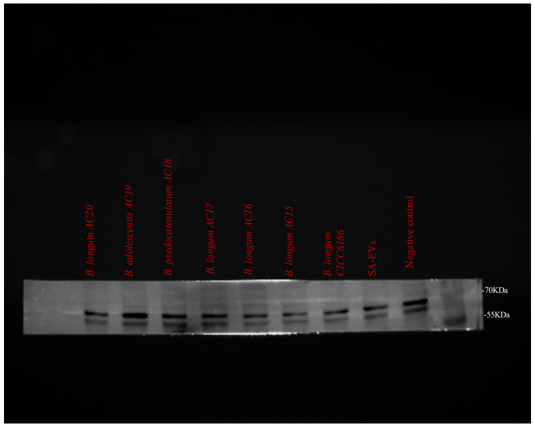

Membrane 1_Beta-actin

Chemi

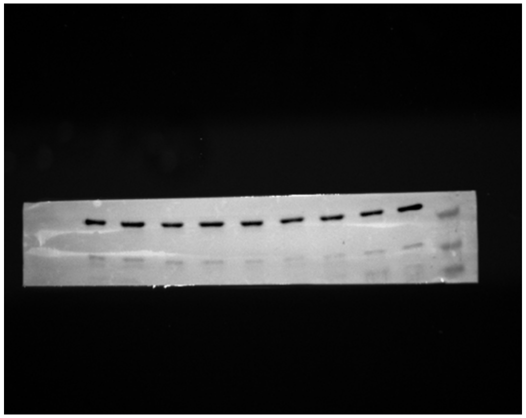

Membrane

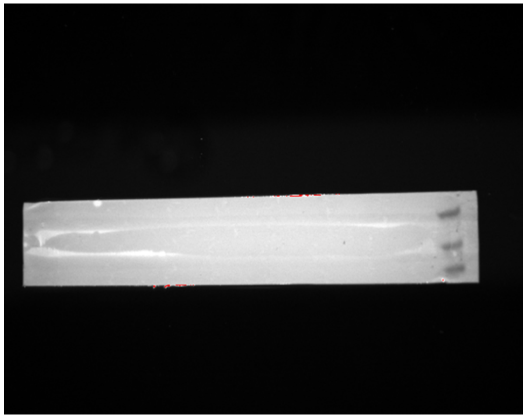

Overlay

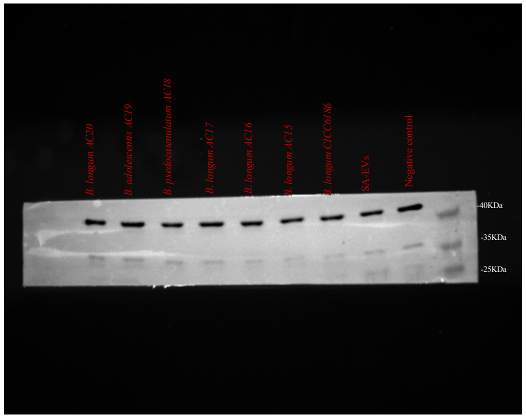

Membrane 2_p65+beta-actin

Chemi

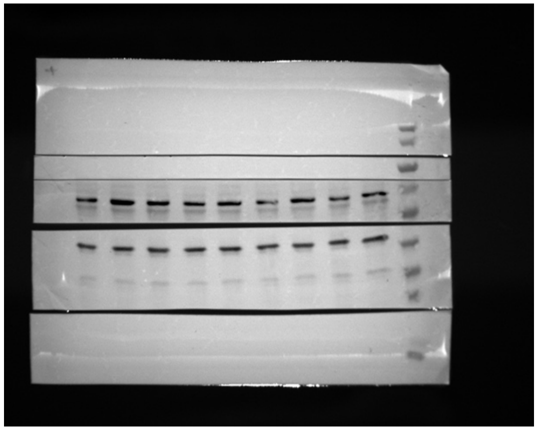

Membrane

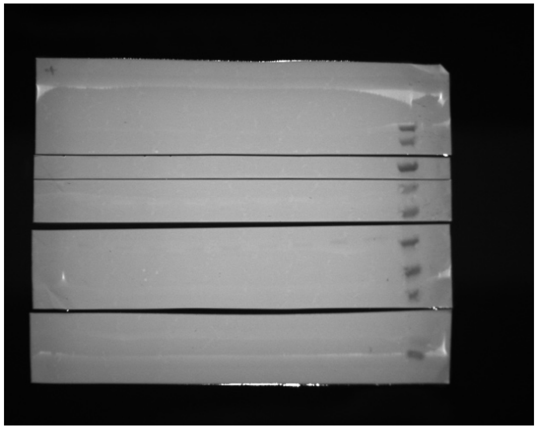

Overlay

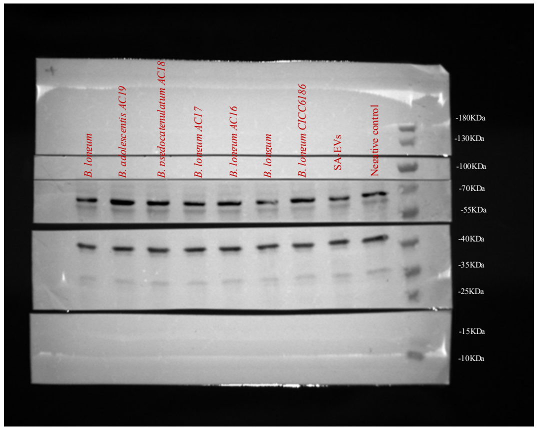

Membrane 2_pp65

Chemi

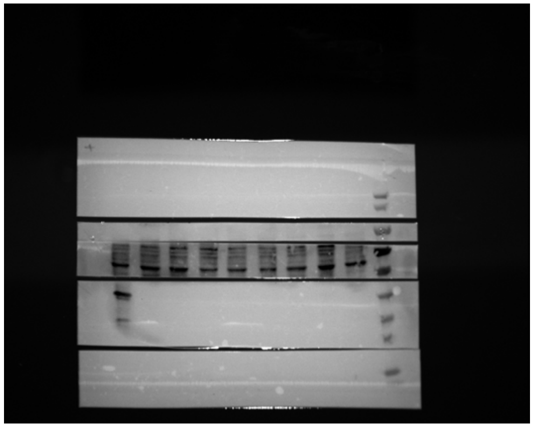

Membrane

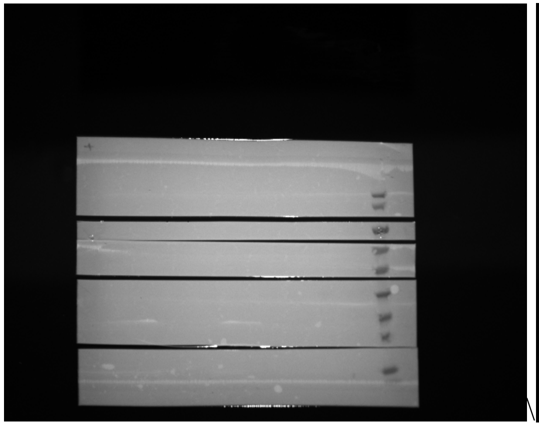

Overlay

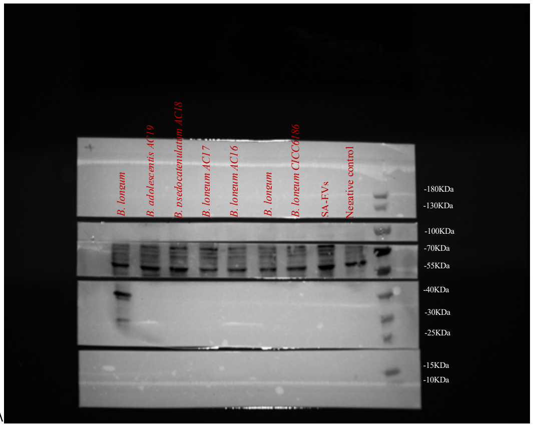

Membrane 2_p65

Chemi

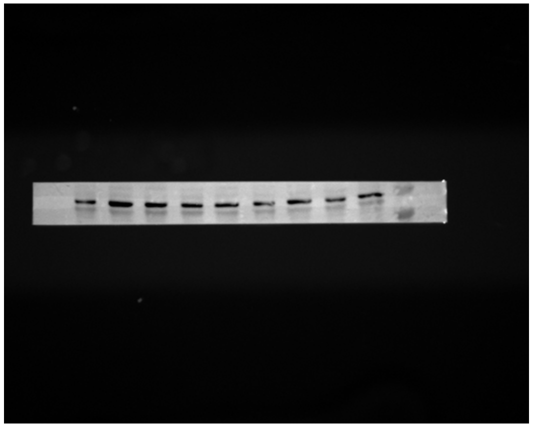

Membrane

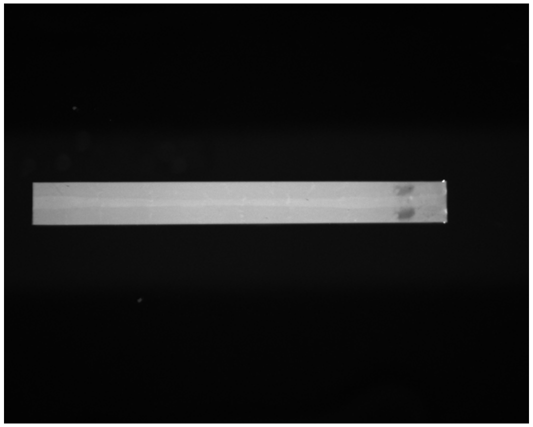

Overlay

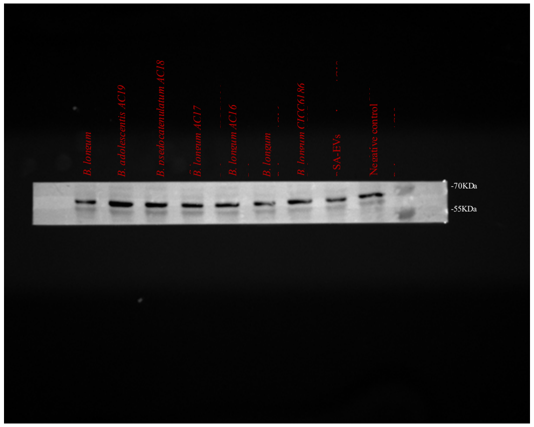

Membrane 2_beta-actin

Chemi

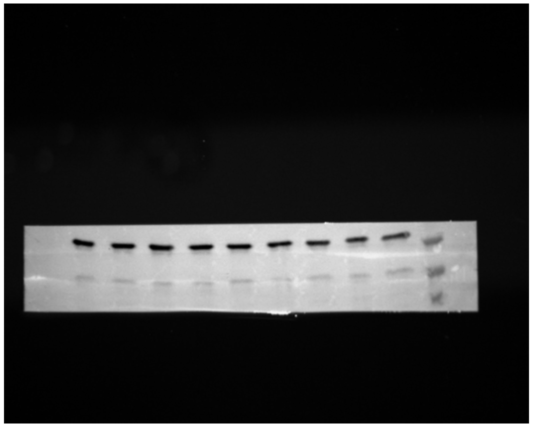

Membrane

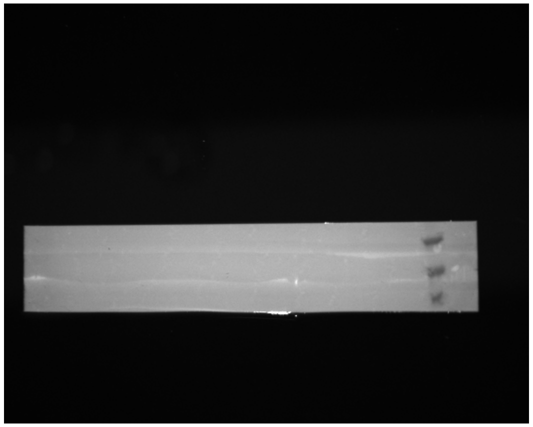

Overlay

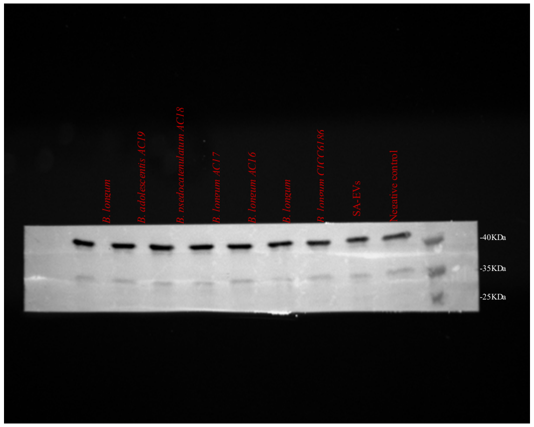

Membrane 3_p65+beta-actin

Chemi

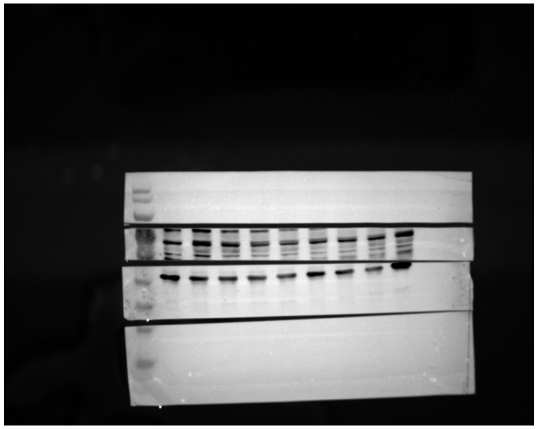

Membrane

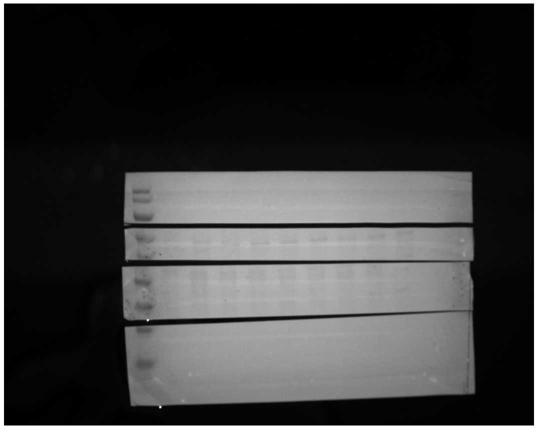

Overlay

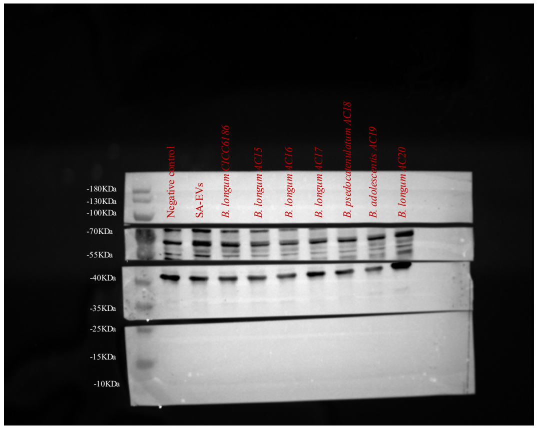

Membrane 3_pp65

Chemi

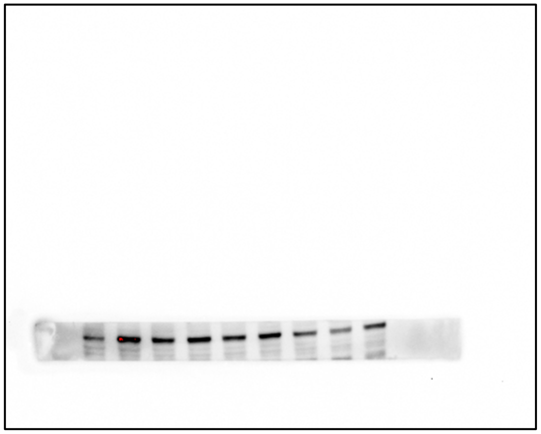

Membrane

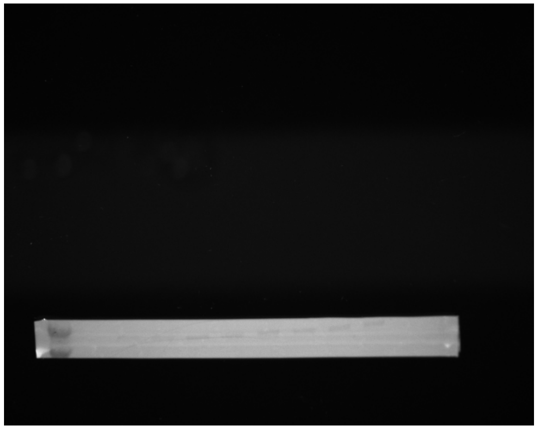

Overlay

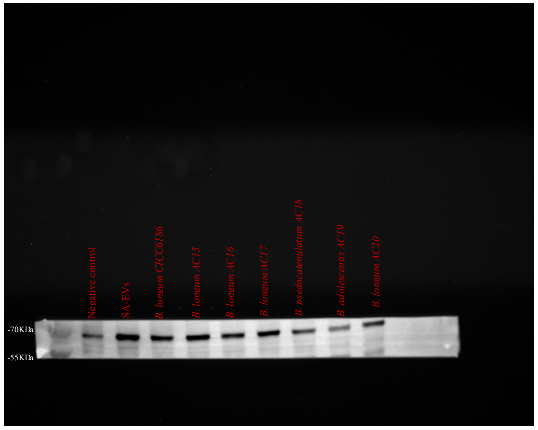

Membrane 3_p65

Chemi | Membrane | Overlay

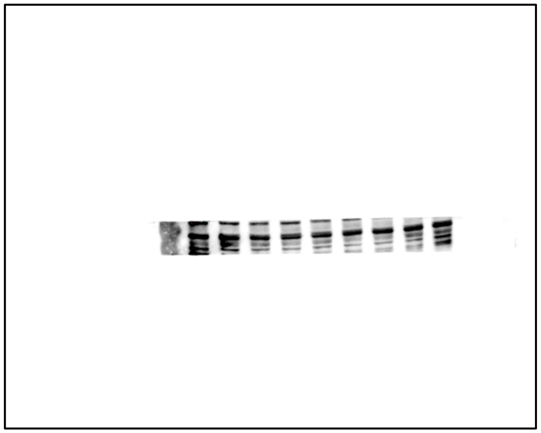

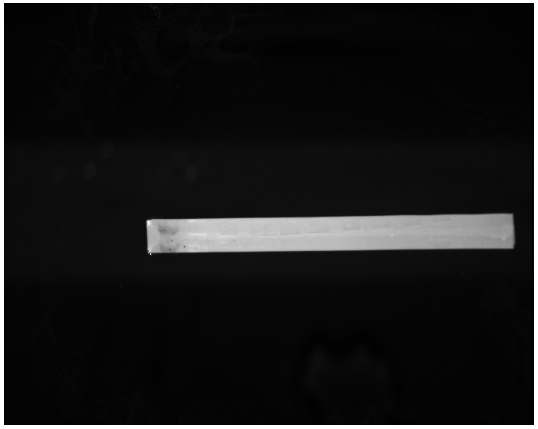

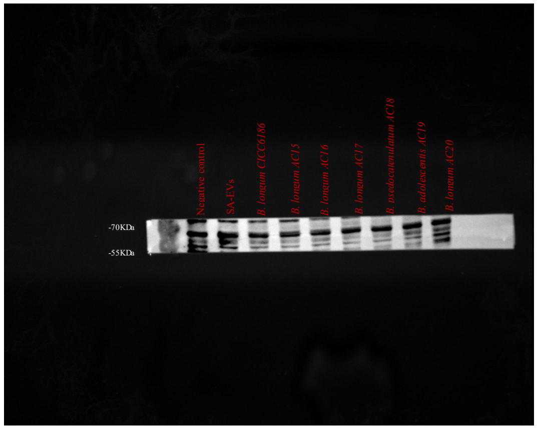

Membrane 3_b-actin

Chemi | Membrane | Overlay

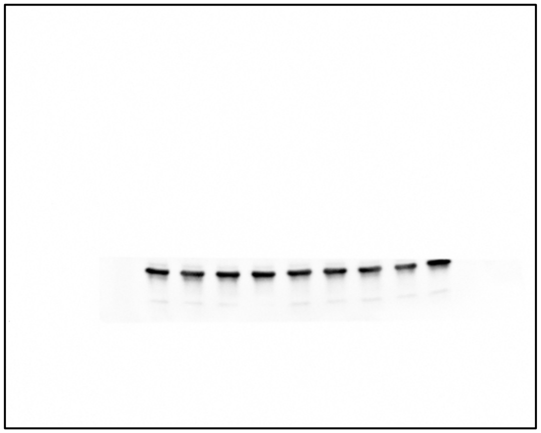

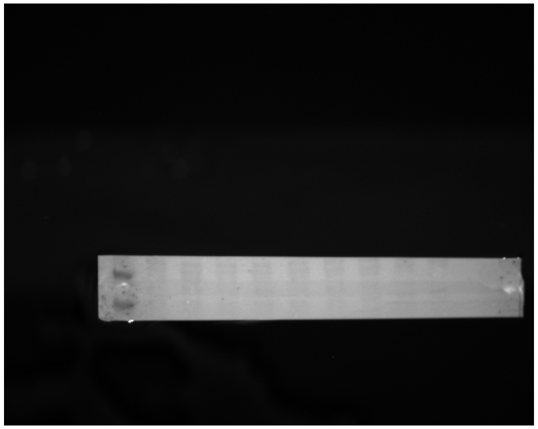

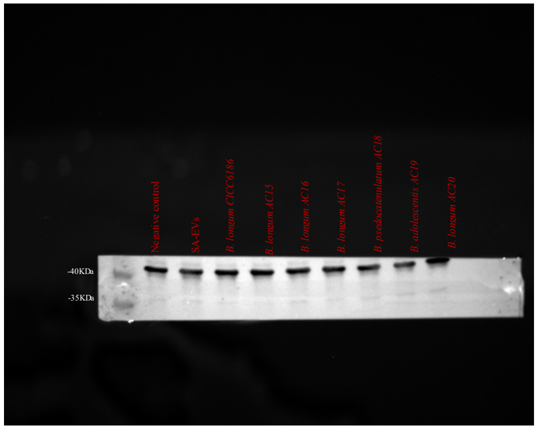

Supplementary Figure 1. The full membrane of western blot analysis the p65 and pp65 of NF-κB signaling pathway on Raw 264.7 cells. All experiments were repeated three times, the expression level of p65 and pp65 were normalized to housekeeping protein, beta-actin.
